# A Potential Interface between the Kynurenine Pathway and Autonomic Imbalance in Schizophrenia

**DOI:** 10.3390/ijms221810016

**Published:** 2021-09-16

**Authors:** Alexandra Büki, Gabriella Kekesi, Gyongyi Horvath, László Vécsei

**Affiliations:** 1 Department of Physiology, Albert Szent-Györgyi Medical School, University of Szeged, Dóm tér 10., H-6720 Szeged, Hungary; buki.alexandra@med.u-szeged.hu (A.B.); kekesi.gabriella@med.u-szeged.hu (G.K.); horvath.gyongyi@med.u-szeged.hu (G.H.); 2 Department of Neurology, Albert Szent-Györgyi Medical School, University of Szeged, Semmelweis u. 6., H-6725 Szeged, Hungary; 3 MTA-SZTE Neuroscience Research Group, H-6725 Szeged, Hungary; 4 Interdisciplinary Excellence Center, Department of Neurology, Albert Szent-Györgyi Medical School, University of Szeged, Semmelweis u. 6., H-6725 Szeged, Hungary

**Keywords:** autonomic nervous system, kynurenic acid, kynurenine pathway, schizophrenia

## Abstract

Schizophrenia is a neuropsychiatric disorder characterized by various symptoms including autonomic imbalance. These disturbances involve almost all autonomic functions and might contribute to poor medication compliance, worsened quality of life and increased mortality. Therefore, it has a great importance to find a potential therapeutic solution to improve the autonomic disturbances. The altered level of kynurenines (e.g., kynurenic acid), as tryptophan metabolites, is almost the most consistently found biochemical abnormality in schizophrenia. Kynurenic acid influences different types of receptors, most of them involved in the pathophysiology of schizophrenia. Only few data suggest that kynurenines might have effects on multiple autonomic functions. Publications so far have discussed the implication of kynurenines and the alteration of the autonomic nervous system in schizophrenia independently from each other. Thus, the coupling between them has not yet been addressed in schizophrenia, although their direct common points, potential interfaces indicate the consideration of their interaction. The present review gathers autonomic disturbances, the impaired kynurenine pathway in schizophrenia, and the effects of kynurenine pathway on autonomic functions. In the last part of the review, the potential interaction between the two systems in schizophrenia, and the possible therapeutic options are discussed.

## 1. Introduction

Schizophrenia is a neuropsychiatric disorder, affecting about 1% of the population, frequently associated with substantial social and economic implications [1]. This generates significant healthcare costs, as central nervous system (CNS) disorders are among the most costly medical conditions (EUR 386 billion annually in the EU) [2]. Schizophrenia is characterized by positive and negative symptoms and cognitive impairments that can influence the patients’ thoughts, perceptions, speech, emotions and behaviors. Positive symptoms include hallucinations, voices that converse with or about the patient and delusions that are often paranoid. Negative symptoms include flattened affect, loss of a sense of pleasure, loss of will or drive and social withdrawal [3]. Additionally, impaired cognitive function is also enduring and core feature with different manifestations [4,5,6].

Besides the behavioral alterations, schizophrenia is commonly associated with autonomic imbalance, that may be related to increased morbidity and mortality [7,8,9,10,11,12,13,14,15,16]. Thus, the autonomic dysfunction is associated with multiple aspects of schizophrenia pathophysiology, including symptom severity, cognitive impairment and the development of cardiometabolic comorbidities, such as metabolic syndrome and high body mass index [8,10,11,14]. The high number of articles (481 in August 2021) found in MEDLINE search using “schizophrenia and autonomic nervous system” (ANS) keywords also suggests that the disturbance of the ANS has a high impact and its treatment is very important in this patient group.

Several neurotransmitter systems are implicated in the neurobiology of schizophrenia, including dopaminergic, glutamatergic, GABAergic (gamma-aminobutyric acid, GABA), adrenergic, cholinergic and serotoninergic [17,18,19,20,21]. Besides the classical neurotransmitters, the kynurenine pathway (KP) metabolites (especially the kynurenic acid [KYNA]) has gained attention in the pathophysiology of schizophrenia. Elevated brain KYNA level, a neuroactive metabolite, is one of the most consistently found biochemical marker in schizophrenia [22,23]. Thus, searching in MEDLINE with “schizophrenia and kynurenic acid” keywords resulted in 270 articles (in August of 2021). KYNA acts as an antagonist of *N*-methyl-d-aspartate and α-7 nicotinic acetylcholine receptors (NMDAR, α7nAChR), both of them involved in the schizophrenia pathogenesis [24,25,26]. Nevertheless, at high concentrations, KYNA can also play a neuroprotective role in conditions of excitotoxicity [27,28,29].

Publications so far have discussed the implication of kynurenines (KYNs) and the alteration of the ANS in schizophrenia independently from each other, only few data suggest that the KP metabolites have effects on autonomic functions [30,31,32]. Nevertheless, the coupling between them in schizophrenia has not yet been addressed, even though their direct common points, potential interaction or similar therapeutic application targets indicate the consideration of their interaction.

**Figure 1:**
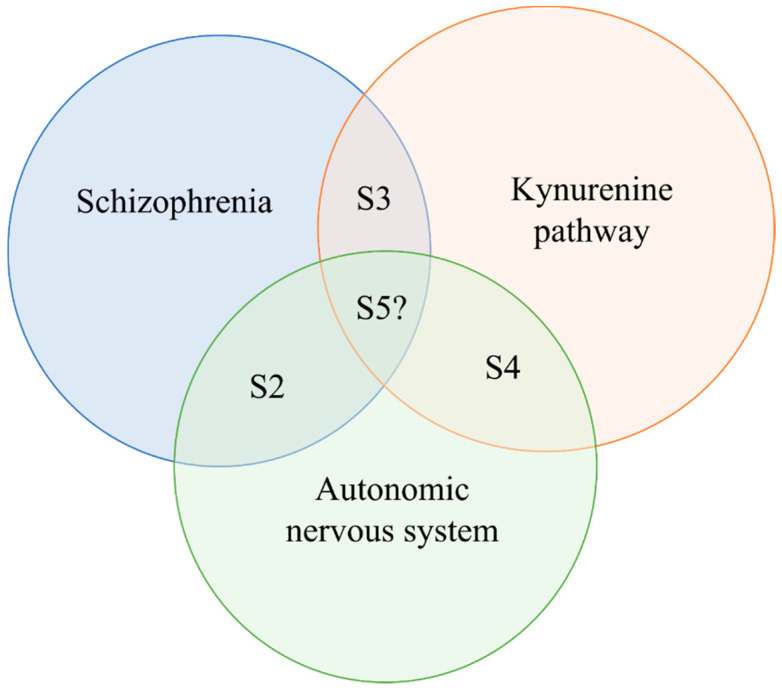
The paradigm of the review. *Abbreviation*: S: section.

The primary aim of this review is to discuss the potential interface between these systems. In Section 2, the alterations of ANS in schizophrenia are characterized (Figure 1). In Section 3, the impaired KP is reviewed in schizophrenia. In Section 4, the effects of KP on autonomic functions are discussed. The Section 5 considers the effects of antipsychotic drugs on these systems, the potential interaction of the ANS and KP in schizophrenia and gathers some novel molecules and therapeutic options along with their targets, which may affect the KP and autonomic functions in schizophrenic patients. Since earlier reviews have discussed the clinical significance of this pathway with regards to psychiatric disorders, this study focuses shortly on this topic in the last part of the review [29,33,34].

## 2. Autonomic Regulation in Schizophrenia

In 1899, Emil Kraepelin described extensive autonomic alterations in patients with schizophrenia, such as increased heart rate, altered pupillary function, increased sweating and salivation, as well as abnormal thermoregulation (Table 1) [9,35]. Since Kraepelin’s publication, several studies suggested impaired ANS activity of these patients compared to the general population [15,36,37,38]. The majority of the research has clearly implicated that the ANS imbalance is more likely a result of diminished parasympathetic rather than elevated sympathetic activity, so the poor parasympathetic modulation is accompanied by relative sympathetic nervous system dominance [8,11,39,40]. However, few studies have reported conflicting findings in relation to the evidence presented above. While some authors did not find evidence of sympathovagal imbalance, others found an augmented sympathetic activity in schizophrenic patients [10,41,42,43,44], or alterations both in sympathetic and parasympathetic activities [10,45].

The altered ANS functions in patients with schizophrenia might be associated with the diverse physical, mental and/or behavioral symptoms. Furthermore, it contributes to poor medication compliance, worsened quality of life, increased severity of psychotic symptoms and increased mortality (including various causes of death in schizophrenia). The increased risk of premature death in patients with schizophrenia is at two to three times that of the general population, that has a great importance to find a potential therapeutic solutions to improve the autonomic disturbances [14,42,45,69,70,71,72,73,74,75].

### 2.1. Symptoms Related to Autonomic Abnormalities in Schizophrenia

The evaluation of blood pressure and heart rate variability can be used to describe autonomic regulation of the cardiovascular system. Regarding the blood pressure, even though systolic and diastolic blood pressure values were moderately higher in schizophrenic patients, its variability did not differ from controls (Table 1) [39]. The complexity of heart rate variability is indicative of cardiac adaptability, thus, the reduced complexity suggests difficulty in heart rate adaptation in response to environmental stimuli (Table 1) [7,46,47]. In antipsychotic treated patients cardiac autonomic dysregulation and reduced complexity of heart rate modulation have been reported, that highly correlated with the pharmacodynamics of the drug applied [7,46,76,77,78]. Thus, the strongest association of low heart rate variability was noted among schizophrenic patients on antipsychotic treatment with high-affinity muscarinic antagonism (i.e., clozapine, olanzapine and quetiapine), that further signify the autonomic dysregulation [14,79,80,81].

Respiration has a significant effect on heart rate oscillations, too, resulting increased parasympathetic activity. Thus, deep breathing could be considered as the most reliable test of parasympathetic function by using the respiratory peak as a quantitative measure of vagal control [82,83,84,85]. Deep breathing results increased variance and high frequency of heart rate in healthy controls, but significantly reduced response was reported in both patients with schizophrenia and their relatives, indicating diminished autonomic reactivity of cardiac functions (Table 1) [38,85].

The dysregulation of body temperature might be intrinsic to the syndrome of schizophrenia, manifested in altered baseline values, abnormal diurnal variation, and an impaired adaptation to heat and cold stress [48]. The higher skin conductance and the disturbed sympathetic skin responses to innocuous stimuli, reported in schizophrenic patients, also refers to the enhanced sympathetic nervous system activity, which showed correlation with symptom severity and poor functional outcome (Table 1) [44,48,51,52,53,54,55,56,57,58,59,60]. Significantly higher rate of sweating was also reported in schizophrenia patients (Table 1) [62].

Regarding the preclinical studies, Pechnick and George evaluated the thermoregulatory effects of phencyclidine (PCP, a noncompetitive NMDAR antagonist, applied as a schizophrenia model) in adult Wistar rats [49]. While acute administration of PCP produced hypothermia, chronic treatment resulted in prolonged hyperthermia [49,50] (Table 1). In a chronic, complex rat model of schizophrenia (developed by postweaning isolation rearing, subchronic ketamine treatment and selective breeding; named Wisket) higher body temperature during the active phase was accompanied by wider range of its fluctuation compared to control Wistar rats (Table 1) [61].

Pupillography is a non-invasive and cost-effective method to determine autonomic activity, and it is suitable to examine both the sympathetic and parasympathetic functions in the context of schizophrenia, too [8,14,64]. The main sympathetically influenced parameter is the resting pupil diameter, which is significantly increased in patients suffering from schizophrenia [8]. Furthermore, “sluggish” parasympathetic function of the pupil by light stimuli and in darkness after pharmacological manipulation was identified among schizophrenia patients [64]. For instance, to induce a pupillary light response in patients with schizophrenia, a 10-fold increase stimulus intensity was required as compared to controls [12]. Similar changes were found in Wisket rats, in which the pupillary light reflex was studied in two series after sedation (diazepam) or anesthesia (chloral hydrate) [63]. Thus, the initial and minimum pupil diameters were greater, the degree of the constriction was lower, and the flatness of the curve and the total duration of constriction were shorter in the sedated Wisket rats compared to control animals. Chloral hydrate anesthesia prolonged the constriction and redilation processes compared to the sedated animals and blunted the differences between the groups (Table 1) [63].

In schizophrenia patients increased sympathetic modulation within the enteric nervous system was also indicated by significantly increased amount of tachygastria and arrhythmia within the gastric pacemaker activity before and after test meal digestion [62]. Additionally, gastric motility disorders, e.g., delayed gastric emptying or diabetic gastroparesis, have been observed in patients, indicating increased sympathetic modulation [62,65].There is a pilot study, in which a significant correlation has been found between salivary α-amylase level and psychiatric symptoms in schizophrenic patients (Table 1) [66]. Thus, the salivary α-amylase reactivity could serve as a potential marker to measure the sympathetic-adrenal-medullary activity [86,87,88].

The vagus nerve is the chief mediator of the bidirectional communication along the gut-brain axis through cholinergic activation of nicotinic receptors on myenteric neurons [14,89]. Furthermore, ANS regulates the mucosal immune response and can also induce changes in gut microbiome composition and activity [14,89,90]. In turn, sensory afferent neurons of the vagus nerve detect a diverse range of stimuli within the intestines and gut microbiome projecting to the nucleus tractus solitarii (NTS) in the brainstem to initiate autonomic, endocrine and behavioral responses [91]. Thus, it is plausible that dysbiosis in schizophrenia may cause aberrant vagal signaling leading to e.g., cardiometabolic disturbances. Further research is needed to investigate the relationship between autonomic dysfunction and dysbiosis in schizophrenia [14].

Beside enhanced urinary frequency or urgency, incontinence and detrusor hyperreflexia were also reported as common autonomic symptoms in schizophrenic patients [67]. In a rat model of schizophrenia treated with ketamine and housed individually the bladder volume significantly decreased, suggesting also detrusor hyperreflexia (Table 1) [68].

### 2.2. Action Mechanisms of ANS Dysregulation

The precise mechanism of ANS dysregulation in schizophrenia remains unclear, both structural and functional changes might be involved. Largely divergent brain centers, including the different nuclei of hypothalamus and brainstem (e.g., locus coeruleus, LC), are associated with sympathetic and parasympathetic activity [13,92]. The underlying causes are likely to be a combination of “peripheral” and “central” mechanisms either with equal importance or with one playing a predominate role [48].

Although the hypothalamus comprises only 2% of the total brain volume, it is a key integrating center of endocrine, autonomic and behavioral responses and homeostatic balance [93]. The hypothalamus is connected with nearly all other brain area, e.g., various limbic structures, brainstem nuclei, thalamus, the cerebral cortex and the pituitary gland [94]. One of the most important hypothalamic nuclei of the central autonomic network is the paraventricular nucleus (PVN). Other hypothalamic nuclei in this network include the dorsomedial nucleus, the lateral hypothalamic area, the posterior hypothalamic nucleus and the mammillary nucleus [95]. So far only few studies have investigated its structural alterations in schizophrenia with inconsistent findings [96,97,98,99,100,101,102]. Some of them reported larger volume of hypothalamus, paraventricular nucleus and the mammillary bodies in schizophrenic patients compared to healthy controls [96,97,98,99]. However, postmortem investigations found decreased or unchanged volumes of these hypothalamic subregions [100,101,102].

LC neurons produce most of the norepinephrine (NE) released in the brain acting on adrenergic receptors [19,103]. LC projects vastly to most of the brain, including subcortical areas, such as the olfactory bulb and dorsal hippocampus; cortical areas, in particular the prefrontal cortex, and reciprocally to other neuromodulator systems, such as the cholinergic, dopaminergic and serotoninergic systems [19]. In turn, LC receives direct input from the cortex and several subcortical areas including amygdala and hypothalamus, and from giganto- and paragiganto-cellular nuclei [104]. The importance of LC in controlling autonomic function results from both direct projections to the spinal cord and projections to autonomic nuclei in the brainstem. A correlation between LC neuronal and sympathetic activities, as evidenced by parameters such as heart rate, blood pressure and cervical sympathetic tone, has been reported [105]. Thus, LC activation produces an increase in sympathetic activity and a decrease in parasympathetic activity via its projections. Therefore, alterations in LC activity affect the complex patterns of neuronal activity throughout the brain, observed as changes in measures of arousal and autonomic function [106].

Evidence for noradrenergic dysfunctions in schizophrenia have been proposed for decades, indicated by elevated NE level in the CSF, brain and serum of patients [107,108]. Abnormalities in LC itself have also been reported in schizophrenia patients with inconsistent results [109,110]. While in paranoid schizophrenia significantly elevated dopamine-beta-hydroxylase activity (that catalyzes the conversion of dopamine to NE) was observed in LC; in elderly schizophrenic patients its marked decrease was reported compared with age-matched controls [109,110]. These neurobiological impairments in schizophrenia correlate with symptoms; thus, the positive and negative symptoms are consistent with the hyper- or hypoactivity of NE system, respectively.

It has to be claimed that numerous genetic, epidemiological and clinical evidence have suggested that inflammatory pathways are also disrupted in schizophrenia, and individuals with infection, inflammation or autoimmune diseases are more susceptible to schizophrenia [111,112,113,114]. Therefore, it cannot be excluded that at least a part of the observed anatomical and physiological alterations might be due to the inflammatory disturbances.

## 3. Kynurenine Pathway in Schizophrenia

Current evidence shows that the tryptophan (TRP) metabolism, including KP is involved in the regulation of many biological systems that are dysfunctional in psychiatric disorders, such as CNS neurotransmission, immune-inflammatory, endocrine and metabolic systems [32,115]. It is highly desirable to expound on the mechanisms through which the KYNs link with many systems and ultimately influence domains such as emotion, cognition and pain [116]. The KP metabolites may underlie different psychotic and cognitive symptoms via neuromodulation [117], thus, the dysregulation of TRP metabolism has been implicated in the pathophysiology of schizophrenia [118,119,120]. Since KYNA, as one of the most important metabolite of KP, acts upon multiple receptors, its abnormal level may disrupt the balance of neurotransmitter systems, as has been detected in various neurodegenerative and neuropsychiatric disorders [121]. This raises the possibility that KYN metabolites have diagnostic (biomarker) and predictive value for the treatment outcome; furthermore, targeting the KP may emerge as a unique opportunity in the development of novel treatments for neuropsychiatric disorders [32,122,123].

**Figure 2:**
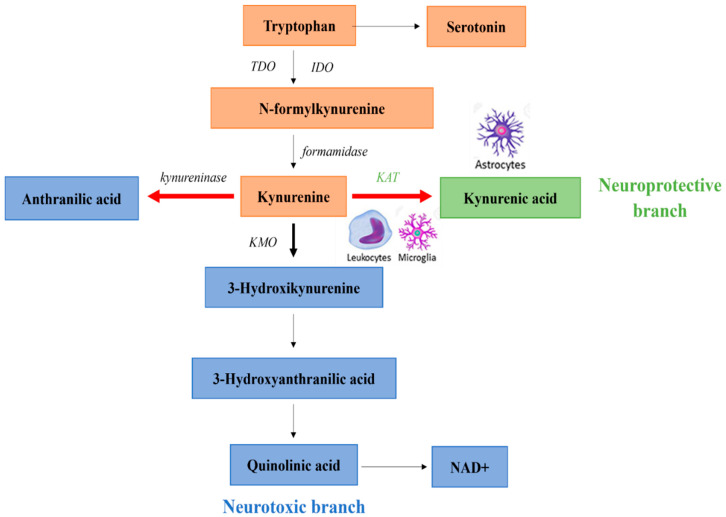
Kynurenine pathway (KP) in normal and schizophrenic subjects. The thick black arrow indicates the primary normal direction of KP. The thick red lines denote shifting in the direction of KP in schizophrenia. *Abbreviations*: IDO: indoleamine 2,3-dioxygenase, KAT: kynurenine aminotransferase, KMO: kynurenine 3-monooxygenase, NAD+: nicotinamide adenine dinucleotide, TDO: tryptophan 2,3-dioxygenase.

The KP accounts for approximately 95% of TRP metabolism and involves several neuroactive metabolites. In the first, rate-limiting step of the KP TRP is oxidized by the following enzymes: indoleamine 2,3-dioxygenase 1 (IDO1), indoleamine 2,3-dioxygenase 2 (IDO2) or tryptophan 2,3-dioxygenase (TDO) (Figure 2). In most tissues, the TRP metabolism is catalyzed by IDO1,2 into *N*-formylkynurenine, then into KYN by formamidase enzyme [124]. IDOs are widely expressed in macrophages, neurons and astrocytes in the brain, and a polymorphism in the allele of IDO rs9657182 has been associated with schizophrenia [125,126,127,128,129,130]. A disbalance in the activation of IDO may be resulted in increased production of KYNA in schizophrenia leading to glutamatergic imbalance [131]. Additionally, proinflammatory cytokines, such as interferon-γ, interleukin 1, and tumor necrosis factor α, which might be enhanced in schizophrenia, are able to shift TRP metabolism to KYN by increasing IDO enzyme activity [114,132,133,134,135].

TDO acting in the brain and liver modulates the available quantity of TRP throughout the body [126]. The enzyme also modulates serotonin levels by reducing the amount of TRP available for synthesis of the neurotransmitter [126,136]. TDO is selectively upregulated in the postmortem frontal cortex of schizophrenic patients [137].

Diverting from the common precursor, KYN, there are two main branches of the KP. In a neuroprotective branch, directly from KYN through irreversible transamination by kynurenine aminotransferases (KATs), KYNA is synthesized in astrocytes and neurons as a terminal metabolite [29,138]. Four subtypes of KATs (KAT1 to KAT4) have been identified, of which KAT1 and KAT2 are thought to have predominant roles in humans [29,139] with the most abundant activities of KAT2 among them (60%). In the other, so called neurotoxic branch of the pathway, guarded by the enzyme kynurenine 3-monooxygenase (KMO), 3-hydroxykynurenine (3-HK) is produced from KYN [114,140]. KMO is expressed either peripherally (kidney and liver) or in the brain [141]. Within the CNS, KMO is primarily expressed in the microglia cells, linking the enhanced production of KMO to inflammatory pathways [138,141,142] 3-HK is further metabolized to 3-hydroxyanthranilic acid by kynureninase that is ultimately leads to the formation of the intermediate quinolinic acid (QUIN). Subsequently, it is transformed to nicotinamide adenine dinucleotide (NAD+), which participates in basic cellular processes [29].

The role of KP disruption in schizophrenia has already been established [119,120,143]. The KYN hypothesis of schizophrenia is built upon the fact that the excess amount of KYNA can induce abnormal behavior by interfering with glutamatergic and cholinergic transmissions, and indirectly affecting dopaminergic signaling, thereby leading to schizophrenic symptoms [25,123,144,145,146,147]. KYNA can influence different types of receptors, possesses broad-spectrum competitive antagonist of all three ionotropic glutamate receptors. It has a stronger affinity for NMDARs, but weaker antagonistic effect on kainate and α-amino-3-hydroxy-5-methyl-4-isoxazole propionic acid receptors (KAR, AMPAR) (Table 2). In studies using human postmortem brain samples, altered expression of NMDARs, AMPARs and KARs have been found in schizophrenic patients, suggesting that the abnormal glutamate neurotransmission is associated with the pathophysiology of the disorder [148,149,150]. Data suggest that KYNA is also an α7nAChR antagonist [144,151,152,153] (Table 2), however, a recent review claims that KYNA does not directly affect nicotinic receptors [154,155].

KYNA has an agonistic effect on aryl hydrocarbon receptor (AHR), a transcription factor involved in the metabolism of xenobiotics [30,114,173,174,175], in the regulation of several physiological processes, including intestinal homeostasis, development, behavior and immunological responses [176,177]. A postmortem human proteomic study identified disrupted AHR signaling in hippocampus of schizophrenic patients [158]. At last, KYNA has been shown to possess agonist activity at an orphan G-protein-coupled receptor (GPR35), which predominantly detected in immune cells and the gastrointestinal tract [30,32,164,165], but no data is available about a link between the GPR35 function and the pathophysiology of schizophrenia.

The reports about the affected KP have confirmed elevated KYNA level in schizophrenia, that seems to be one of the most consistently found biochemical aberrations in this disease [23,178,179,180,181]. Indeed, the higher level of brain KYNA concentration is in connection with the higher degree of cognitive impairment in schizophrenia [24,182]. Additional insight can be derived from the findings of recently published meta analyses, which determined the central and peripheral KP metabolites in schizophrenia [183,184]. The KYN is also elevated in both CSF and cortical brain regions, but the neurotoxic branch of the KP seems to be unaffected, since QUIN and 3-HK were found at normal levels in the postmortem schizophrenic brain [119,178,180,181,185]. Studies investigating peripheral serum levels of KYN, 3-HK, KYNA and QUIN detected no difference between first-episode neuroleptic-naive patients and controls [183,186,187]. Whereas another study suggested that enhanced serum level of 3-HK could predict the symptom severity in schizophrenia [188]. In addition, another metabolite of the KP, anthranilic acid, is also markedly increased in serum of schizophrenia patients [189].

Further elements related to KP, including enzymes, are also altered in schizophrenia. The genetic variants of these enzymes, leading to the abnormal level of KYNA, have been linked to psychotic symptoms and cognitive dysfunctions [190,191,192]. In postmortem brain samples of schizophrenic patients reduced KMO activity has been reported [192]. The increased KYNA production and level may be directly related to increased KAT activity (Figure 2). In support of this finding, enhanced number of astrocytes, as the primary source of KYNA, has been found in patients with schizophrenia [186,193]. However, in a postmortem tissue analysis of schizophrenic patients revealed an increase in KYNA that was associated with decreased KMO activity rather than alterations in KAT activity [181,194]. The TDO activity is also upregulated in the postmortem brains of schizophrenic patients [137,192].

Animal models also support the KYN hypothesis of schizophrenia [163,169,171,195]. Thus, similarly to the NMDA receptor antagonists, experimentally increased brain concentration of KYNA induces impairments in cognitive flexibility, memory, long-term potentiation and sensory gating, as well as increased amphetamine-induced locomotor activity and disturbed social interaction and reward circuitry were detected [157,161,162,163,164,167,168,169,170,171,172]. These effects might be due to the down-regulation of the permissive action of α7nAChRs, since the antagonism of α7nAChR by KYNA may induce psychotic symptoms [156,157].

In the last decade, a growing number of evidence proved the role of the interaction between inflammation and KP in the pathophysiology of schizophrenia [117]. The inflammatory mediators can unbalance the KP in schizophrenia and the resulted KP metabolites can, in turn, underlie different psychotic symptoms via modulation of neurotransmission [196].

The conventional view is that peripheral KYNA and QUIN poorly cross the blood-brain barrier (BBB), thus, these metabolites may be derived in situ from KYN in the brain. Therefore, it was suggested that they may not necessarily play important role in the pathogenesis of schizophrenia [196]. In contrast TRP, KYN, and 3HK can be transported across the BBB [32,197]. Under physiological conditions 60−80% of KYN in the brain is exogenous origin, being actively transported across the BBB by the large neutral amino acid transporter 1 [32]. The neuroinflammation in neuropathology of schizophrenia is closely associated with the disruption of the BBB integrity due to microglial activation and elevated cytokine production, such that the entry of circulating KP metabolites into the CNS can be elevated [198,199,200,201,202]. Following systemic immune activation the transport of the KYN may approach 100%, although if inflammation is limited to the brain, KYN can be produced centrally from TRP [203].

It should also be mentioned that KYNA, as a potent antioxidant metabolite, at higher concentrations can exerts neuroprotection, thus, the clinical significance of KP abnormalities in patients with schizophrenia remains unclear [204]. Thus, the elevation of brain KYNA level, either by administration of L-KYN or pharmacological manipulation of the availability of the KP enzymes, has become an attractive strategy to attenuate neuroinflammatory responses and to protect against glutamate induced excitotoxicity associated with ischemic brain injury [29,205,206]. Furthermore, during neuroinflammation both in the periphery and in the brain, KYNA may produce anti-inflammatory responses through the activation of AHRs [30,114,173,174,175].

In summary, it should be claimed that the role of KP in the etiology of schizophrenia is complex and the literature contains numerous inconsistent findings. Alongside this, physiological variations in patients may occur within different schizophrenic subgroups [196].

## 4. The Interplay of Kynurenines and Autonomic Functions

**Figure 3:**
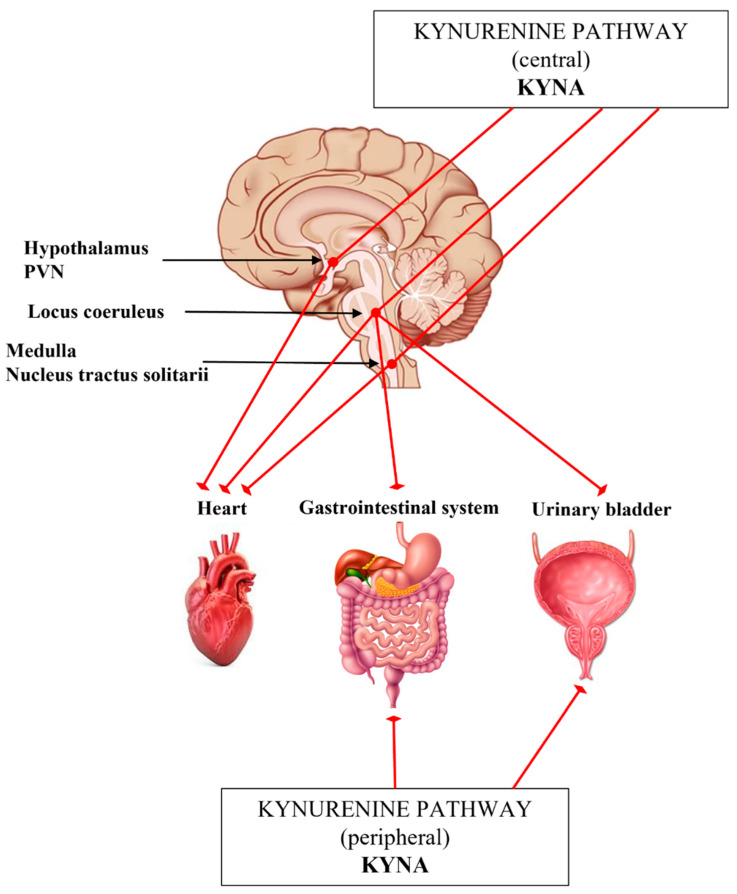
Schematic representation to the Section “The interplay of kynurenines and the autonomic functions”. *Abbreviations*: KYNA: kynurenic acid, PVN: paraventricular nucleus.

Some preclinical studies suggest that KP metabolites have significant role in the regulation of the ANS, including cardiovascular, gastrointestinal systems and urinary tract (Table 3) [159,207,208]. However, we still do not know exactly, how the KYNA and/or other KP metabolites are involved in the modulation of sympathetic outflow. The results of studies investigating stress-related TRP metabolism supported that, in part through the activation of the hypothalamic–pituitary–adrenal (HPA) axis, including the sympathetic nervous system, the formation of KYNA and other KP metabolites are stimulated [22,209,210,211,212,213]. The increased extracellular concentration of KYNA in rat brain has been associated with reduced cell firing rate in LC, presumably, the endogenous KYNA counterbalances the effects of glutamate (Figure 3) [106,214]. Thus, KYNA is claimed to be a hypotensive agent in rats [215,216]. Microinjection of KYNA into the PVN promoted decreased mean arterial blood pressure and heart rate [217] (Figure 3, Table 3). In the ventral part of the medulla and in the NTS many KAT-immunoreactive neurons were found in association with NMDA receptors involved in the control of blood pressure [218]. In agreement with it, glutamate injected into the rostral ventrolateral medulla elevated the blood pressure and heart rate in anesthetized rats, but the centrally administered KYNA lowered the blood pressure (Figure 3, Table 3) [219,220].

In other research, in spontaneous hypertensive rat strain, developed by a missense mutation of KAT-I gene, with abnormally low KYNA levels in the medulla were linked to high blood pressure, which was dose dependently attenuated by L-KYN (Table 3) [221,222,223]. Furthermore, KYNA microinjection into the NTS in awake rat blocked the processing of the parasympathetic component (bradycardia) of the chemoreflex, however, the pressor response was only partially reduced [224] (Figure 3). Similar results were seen in a working heart-brainstem preparation of rats [230].

Some data suggest that the gastrointestinal function is also influenced by the KP. Thus, the activation of LC neurons by colon distension was significantly antagonized by intracerebroventricular (icv.) administration of KYNA (Table 3) [207]. Gastrointestinal motility regulation is predominantly cholinergic in nature, but NMDARs are also present and abundantly expressed on enteric cholinergic neurons [231,232,233,234,235,236]. Glutamate or its endogenous receptor agonists/antagonists may participate in modulation of the enteric cholinergic function, since NMDAR activation enhances the nitrogen monoxide dependent acetylcholine release from the myenteric neurons in the ileum and colon (Figure 3) [237,238,239]. KYNA and SZR72 treatment decreased the motility index, nitric oxide synthase activity and reactive oxygen species in intestinal obstruction and colitis-induced models [33,225,226,227].

The KP also has effects on the urinary tract (Figure 3, Table 3). Anatomical and electrophysiological studies have demonstrated the existence of glutamate input from the ventrolateral medulla to the LC. The icv. administered KYNA could prevent both increases in LC discharge rate and EEG activation elicited by bladder distention and inhibited the urinary bladder contractions evoked by stimulating the pontine micturition center [228,229]. In a recent study a potential treatment of hyperreflexic urinary bladder and detrusor-sphincter dyssynergia have been investigated in a rat model [208]. The administration of replication-defective herpes simplex virus vectors encoding the KAT2 gene into the bladder wall blocked NMDARs in the lumbosacral dorsal root ganglia, as well as in the lumbosacral micturition center through the enhanced production of KYNA (Figure 3) [208].

## 5. The Potential Interaction of the ANS and KP in Schizophrenia, and Molecules Mutually Targeting KP and Autonomic Dysfunctions in Schizophrenia

It is important to mention that antipsychotic drugs may produce adverse effects on autonomic functioning and sympathovagal balance [11,14,240,241]. The extent to which they exacerbate autonomic dysfunction in schizophrenia highly depends on the pharmacodynamics of the antipsychotic taken [14]. These disturbances appear mainly in the cardiovascular system with a negative correlation between neurocardiac control and the degree of antipsychotic affinity and antagonism of muscarinic receptors (M1–M5). Furthermore, antipsychotics with high muscarinic affinity (olanzapine, clozapine and quetiapine) show greater reductions in heart rate variability, than low muscarinic affinity antipsychotics, like risperidone or aripiprazole [242,243,244]. Additionally, in cases of olanzapine and clozapine, their antiadrenergic properties also play a role in ANS modulation. For example, olanzapine, as an antagonist of α1 adrenergic receptors, causes vasodilation and reduction in blood pressure, which thereby initiates a reflexive sympathetic response. Similarly, clozapine enhances the noradrenergic activity through the antagonism of α2 adrenergic receptors [245]. Indeed, studies examining catecholamine levels have revealed that clozapine significantly increases plasma NE [14,246].

Treatment options based on targeting the KYN metabolism in schizophrenia may influence the autonomic signs discussed in this section, although no direct evidence is available for the complex interaction between these systems. Regarding the complex interplay of the ANS, KP and schizophrenia (S4), only few indirect data are available.

As the inflammatory hypothesis of schizophrenia is supported by clinical studies and the KP is associated with inflammatory processes, thus the schizophrenia-related neuroinflammation could increase KMO-dependent KYN metabolism, leading to the accumulation of 3-HK and QUIN [34,130,247,248]. In schizophrenia the blunted type-1 immune response with various inflammatory stimuli in microglia may modify the rate-limiting step of the KP. Thus, TRP is catalyzed by IDO into N-formyl-L-kynurenine in the hypothalamic, pituitary and adrenal areas, influencing the function of ANS [29,249,250]. The induction of IDO could potentially result in production of toxic metabolites (3-HK, QUIN), therefore, CNS infections or other inflammatory processes could negatively affect the function of the HPA axis through interferon-γ induced upregulation of IDO [250]. Furthermore, decreased serum level of 3-HK following antipsychotic therapy was detected [145].

It is suggested that several ways for the improvements of autonomic signs might be available through influencing the KP in schizophrenia. A review written by Dounay et al. highlights recent advances in medicinal chemistry toward three of the enzyme targets in KP: IDO1, KAT’s and KMO [141], and these enzymes have currently the greatest potential as drug targets for preclinical and clinical investigation of the KP. Thus, numerous medicinal chemistry studies are aimed to design novel, potent and selective inhibitors for each of these enzymes [141].

### 5.1. Direct Modulators of the KP

In ketamine-induced schizophrenia rat model the IDO inhibitor 1-methyl-D-tryptophan and the TDO inhibitor allopurinol prevented lipid peroxidation and increased superoxide dismutase and catalase activity in brain region specific manner (Table 4) [130]. Moreover, in a depression mice model glycyrrhizic acid prevented the activated enzymes in KP and the development of depressive-like behaviors [251] Therefore, it was suggested that these KP inhibitors could represent a viable therapeutic target in treating schizophrenia and other diseases associated with neuroinflammation and oxidative stress. The regulatory region of the tdo2 gene, containing glucocorticoid response elements, could be upregulated by dexamethasone, an anti-inflammatory glucocorticoid drug [130,252,253,254]. Thus, the expression of TDO is induced by glucocorticoid hormones, glucagon, estrogens, and also regulated by the availability of its substrate, TRP [126,136,255,256,257,258,259,260]. Moreover, there are reports indicating that TDO activation is stimulated by free radicals. It is suggested that the inhibition of IDO and TDO enzymes promote antioxidant effect via the inhibition of the production KYN metabolites [130]. Co-treatment with antidepressant tianeptine (specifically enhancing serotonin uptake), the allopurinol improved the chronic stress induced depressive-like behavior in rats (Table 4) [261]. However, no potential TDO selective inhibitor has entered clinical trials thus far [261].

Drugs influencing the KAT enzymes are one of the most intensely studied therapeutic approach in schizophrenia [272]. Previous electrophysiological and behavioral studies approaching KAT2 inhibition have confirmed their beneficial effects [114,273,274,275,276,277,278,279,280]. Clinical trials showed that D-cycloserine, has positive effects on cognitive functions in patients with schizophrenia or those experiencing Alzheimer’s delusions, by decreasing KYNA levels in the brain by inhibiting KAT1, 2 and 3 [262,263,272]. It has been reported that administration of a systemically available KAT2 inhibitor, PF-04859989, restores glutamate release events in the prefrontal cortex of rats exhibiting elevated KYNA levels [264]. It has been published that angiotensin receptor blockers inhibit KAT2 activity and reduce KYNA production in rat cortical slices (Table 4) [215].

Nowadays, the research is focused on KMO inhibitors to reduce QUIN synthesis thereby reducing neural activity and excitotoxicity in neurodegenerative disorders [281]. Thus, specific KMO inhibitors (e.g., Ro 61-8048) resulted in anticonvulsive, neuroprotective and anti-dyskinetic effects leading to the acceptance of KMO as a drug target (Table 4) [268,282,283,284,285,286]. On the other hand, an interruption of KMO activity would favor a metabolic shift towards the production of KYNA [194]. Nonetheless, pharmacological inhibition of KMO might also lead to undesirable consequences on the nervous and immune systems that will have to be evaluated carefully in animals prior to clinical studies, therefore KMO inhibitors are still at the pre-clinical phase of development [120,276].

### 5.2. Indirect Modulatory Options of the KP

As was mentioned above, the BBB integrity is decreased in schizophrenia due to enhanced inflammatory processes, thus a promising treatment strategy might be based on reducing the access of circulating KYN to the brain. In this vein, it has been demonstrated that leucine treatment in mice is a potential method of competitively blocking the large neutral amino acid transporter 1 to prevent systemic KYN from entering into the brain and attenuate the formation of neurotoxic KYN metabolites (Table 4) [198].

Some studies suggested that the parasympathetic activation through vagal nerve stimulation (VNS) inhibited cytokine production, improved heart rate variability, and ameliorated low moods and emotional symptoms in depressive patients resistant to pharmacological treatment [269,270]. This method is referred as a promising add-on treatment for treatment-refractory depression, but its application in other psychiatric disorders, such as dementia or schizophrenia is increasingly evaluated (Table 4) [287]. Recent studies have indicated that VNS controls both peripheral and central inflammation via α7nAChRs [247]. Since KYNA, as a potent non-competitive α7nAChR-antagonist, facilitates disturbances in schizophrenia, the VNS might have a counterbalancing effect [144,247].

Adenosine, an endogenous nucleoside formed by the degradation of adenosine-triphosphate (ATP) during energy-consuming processes, acts as a neuromodulator. It influences multiple physiological processes through activation of four subtypes of G-protein coupled membrane purinergic receptors (A1, A2A, A2B and A3) controlling synaptic plasticity and neurotransmitter release (e.g., glutamate, dopamine and GABA [288,289,290,291]. Of relevance to this review adenosine acts on brainstem nuclei involved in autonomic cardiovascular regulation. In general, it decreases sympathetic tone through complex and incompletely understood mechanisms of action [290,291]. The “adenosine hypothesis of schizophrenia” postulates that a reduced adenosinergic tone is involved in the dysregulation of glutamatergic and dopaminergic activity in schizophrenia patients [292,293,294,295,296,297,298,299]. The dopamine level in schizophrenia is, at least partly, under the control of adenosine: reduced activation of presynaptic A1 receptors can trigger increased availability of dopamine leading to increased basal activity of the dopamine D2 receptor, thus promoting psychosis [300,301,302,303]. In addition, in the basal ganglia adenosine directly modulates dopaminergic signaling through complexes formed between adenosine and dopamine receptors [304,305,306,307]. A2A receptors exert fine regulation of individual synapses and their activation facilitates glutamate release and potentiates NMDAR function [294,308]. Therefore, A2A receptors regulate synaptic plasticity by promoting adequate (or aberrant) adaptive responses in neuronal circuits [294,300,309]. Microinjection of adenosine into the NTS evokes a dose-dependent decrease in blood pressure, heart rate, and renal sympathetic nerve activity, primarily through A2A receptors (Table 4) [310]. That is also the case for the A3 receptor regulating serotonergic and glutamatergic systems [311,312]. A link between adenosine hypofunction and schizophrenia is supported by clinical evidence demonstrating increased enzymatic degradation of adenosine in patients [313,314]. Therefore, this neurotransmitter system might be a potential target for novel drug treatment of several psychiatric conditions, including schizophrenia [315]. An open clinical trial demonstrated that patients treated with a combination of haloperidol and dipyridamol (an adenosine uptake inhibitor, increasing adenosine availability in the synaptic cleft) had a greater improvement in positive symptoms compared with patients treated with haloperidol alone (Table 4) [271,293]. Accordingly, based on informative preclinical studies, adenosine receptor agonists may act as atypical antipsychotic drugs implicating for the proposed use of them in schizophrenia treatment [294,316]. Although neurobiological properties of adenosine may be linked to KYNA, interactions between the adenosinergic system and the KP have not been carefully examined so far. However, both of them are associated with schizophrenia, thus a deeper understanding of their interactions may lead to the development of innovative strategies for the treatment of this disorder [292]. Specifically, the effects of combined approaches with adenosine receptor ligands and compounds able to reduce brain KYNA levels (e.g., KAT 2 inhibitors) have not been assessed experimentally so far. These studies might support the development of new multi-target therapeutic strategies that focus on both the purinergic system and KP.

Unfortunately, it should be claimed that none of the above-mentioned treatment options were investigated regarding the autonomic dysfunction of schizophrenia and KP.

## 6. Summary and Conclusions

There is an established correlation between schizophrenia and autonomic dysregulation, and almost all function of the autonomic nervous system is affected in schizophrenic patients. The investigation of the ANS function in individuals who are at increased risk of developing schizophrenia may be particularly useful to identify the dysregulated physiological patterns at an early stage of schizophrenia, and to develop appropriate interventions [10].

The KYNA hypothesis of schizophrenia is built upon the fact that the elevated KYNA interferes with disturbed glutamatergic and cholinergic transmission [25,123,144,145,146,147]. Furthermore, metabolites of the KP can influence the functionality of the autonomic system (see Section 4). Therefore, it might be assumed that an overlap between these systems might have a significant role in the etiology of schizophrenia, however, extensive studies are required to adequately explore this hypothesis. Though the treatment of schizophrenia remains challenging, a better understanding of the interplay between KP and ANS could introduce novel therapeutic options and considerations for drug treatments. Furthermore, it should be mentioned that drugs influencing the KP and/or ANS might be important tools for decreasing antipsychotic-induced adverse effects.

Alterations in KP and ANS are present not only in schizophrenia, but also in other neurological disorders, such as migraine, or neurodegenerative disorders including Huntington’s, Alzheimer’s, Parkinson’s diseases and multiple sclerosis [261,317,318,319,320,321,322,323]. Studying the interaction between ANS and KP in different neuropsychiatric disorders might broaden our knowledge to understand mechanisms and disorders apart from schizophrenia and help to find new therapeutic solutions.

## Figures and Tables

**Table 1:** Autonomic nervous system related clinical and preclinical findings in schizophrenia.

ANS Assessment	Clinical Findings	References	Preclinical Findings	References
**Blood pressure**	Moderately higher systolic and diastolic blood pressure	[39]		
**Blood pressure variability**	No changes	[39]		
**Heart rate variability**	Reduced complexity	[7,46,47]		
**Thermoregulation**	Abnormal diurnal variation of body temperature	[48]	PCP rat model: Acute PCP: hypothermia Chronic PCP: hyperthermia Perinatal PCP: long-lasting effect on the baseline temperature	[49,50]
	Impaired ability to heat stress adaptation Higher skin conductance Disturbed sympathetic skin responses	[44,48,51,52,53,54,55,56,57,58,59,60]	Wisket rat model: Higher body temperature, Higher alteration rate	[61]
	Higher rate of sweating	[62]		
**Pupil function**	Increased resting pupil diameter	[8]	Wisket rat model: Sedation—several parameters disturbed Anesthesia—prolonged constriction and redilation processes, blunted differences	[63]
	A “sluggish” parasympathetic function by light	[64]		
	Decreased stimulus sensitivity	[12]		
**Gastrointestinal system**	Increased tachygastria, arrhythmia	[62]		
	Delayed gastric emptying, diabetic gastroparesis	[62,65]		
	Correlation between salivary α-amylase level and psychiatric symptoms	[66]		
**Urinary system**	Enhanced urinary frequency, urgency, incontinence and detrusor hyperreflexia	[67]	Wisket rat model: decreased bladder volume	[68]

*Abbreviations*: ANS: autonomic nervous system, PCP: phencyclidine.

**Table 2:** Receptor-binding profile of KYNA.

Receptor	Activity	References
**α7nAChRs**	antagonist	[154,155,156,157]
**AHR**	agonist	[158]
**AMPAR**	antagonist	[148,149,150]
**GPR35**	agonist	[30,32,159,160]
**KAR**	antagonist	[149,150]
**NMDAR**	antagonist	[121,149,150,157,161,162,163,164,165,166,167,168,169,170,171,172]

**Table 3:** The interplay of kynurenines and the autonomic functions.

ANS Assessment	Preclinical Findings	References
**Hypothalamus**	HPA axis stimulates the formation of KYNA and other KP metabolites	[22,209,210,211,212,213,217]
	KYNA in the PVN decreased mean arterial blood pressure and heart rate	[217]
**Brainstem**	L-KYN decreases the elevated blood pressure	[219,220,221,222,223]
	Chemoreflex: KYNA blocked the parasympathetic component (bradycardia), pressor response was reduced	[224]
**Gastrointestinal system**	Activated LC by colon distension antagonized by administered KYNA	[207]
	KYNA decreased the motility index Colitis model: KYNA and SZR-72 decreased the motility index, normalized the smooth muscle tone, had an inhibitory effect on the colitis-induced high reactive oxygen species activities	[33,225,226,227]
**Urinary system**	KYNA prevented LC elicited by bladder distention KYNA inhibited the urinary bladder contractions evoked by stimulating the pontine micturition center	[228,229]

*Abbreviations*: HPA: hypothalamic–pituitary–adrenal, KYNA: kynurenic acid, LC: locus coeruleus, L-KYN: L- kynurenine, KP: kynurenine pathway, PVN: paraventricular nucleus.

**Table 4:** Some therapeutic options may influence the KP.

Molecule/Therapy	Action Mechanism	Effect	Preclinical Model	Ref.	Clinical Study	Ref.
**1-methyl-d-tryptophan**	IDO inhibitor	Decreased neuroinflammation and oxidative stress	Ketamine-induced schizophrenia rat model	[130]		
**Allopurinol**	TDO inhibitor	Antioxidant effect	Ketamine-induced schizophrenia rat model	[130]		
		Improved depressive-like behavior	Chronic stress-induced depression mice model	[261]		
**d-cycloserine**	KAT inhibitor	Improved cognitive functions			Schizophrenia	[262,263]
**PF-04859989**	KAT inhibitor	Restored glutamate release events	Rat model exhibiting elevated KYNA levels	[264]		
**Angiotensin receptor blockers**	KAT inhibitor	Reduced KYNA production	Rat cortical slice	[215,265,266,267]		
**Ro 61-8048**	KMO inhibitor	Neuroprotection	Gerbil and rat model of brain ischemia	[268]		
**Leucine**	Large neutral amino acid transporter 1 blocker	Prevented systemic KYN from entering into the brain	Depression-like mice model	[198]		
**VNS**		Anti-inflammatory, Improved heart rate variability, low moods and emotional symptoms			Depression	[269,270]
		Anti-inflammatory via α7nAChRs			Schizophrenia	[247]
**Dipyridamol**	Adenosine reuptake inhibitor	Greater improvement in positive symptoms and general psychopathology symptoms			Schizophrenia	[271]

*Abbreviation*: VNS: vagal nerve stimulation.

## Data Availability

Not applicable.

## Abbreviations

α7nAChR	α-7 nicotinic receptors acetylcholine
AHR	aryl hydrocarbon receptor
AMPAR	α-amino-3-hydroxy-5-methyl-4-isoxazole propionic acid receptor
ANS	autonomic nervous system
BBB	blood-brain barrier
CNS	central nervous system
CSF	cerebrospinal fluid
GABA	γ-aminobutyric acid
GPR35	G-protein-coupled receptor 35
3-HK	3-hydroxykynurenine
HPA	hypothalamic–pituitary–adrenal
IDO	indoleamine 2,3-dioxygenase
icv	intracerebroventricular
KAR	kainite receptor
KAT	kynurenine aminotransferase
KMO	kynurenine 3-monooxygenase
KP	kynurenine pathway
KYNA	kynurenic acid
KYN	kynurenine
LC	locus coeruleus
NE	norepinephrine
NMDAR	*N*-methyl-d-aspartate receptor
NTS	nucleus tractus solitarii
QUIN	quinolinic acid
PCP	phencyclidine
PVN	paraventricular nucleus
TDO	tryptophan 2,3-dioxygenase
TRP	tryptophan
VNS	vagal nerve stimulation
